# Modulation of the Inflammatory Process by Hypercholesterolemia in Osteoarthritis

**DOI:** 10.3389/fmed.2020.566250

**Published:** 2020-09-25

**Authors:** Amanda Villalvilla, Ane Larrañaga-Vera, Ana Lamuedra, Sandra Pérez-Baos, Alberto G. López-Reyes, Gabriel Herrero-Beaumont, Raquel Largo

**Affiliations:** ^1^Bone and Joint Research Unit, Instituto de Investigación Sanitaria Fundación Jiménez Diaz (IIS-FJD), Universidad Autónoma de Madrid (UAM), Madrid, Spain; ^2^Geroscience Laboratory, Instituto Nacional de Rehabilitación Luis Guillermo Ibarra Ibarra, Mexico City, Mexico

**Keywords:** osteoarthritis, cartilage, hypercholesterolemia, chondrocyte, LDL, diet

## Abstract

**Objective:** Several studies have linked metabolic syndrome to the development of osteoarthritis (OA) through hypercholesterolemia, one of its components. However, epidemiological studies showed contradictory results, and it is not clear how hypercholesterolemia itself, or oxidized LDL (oxLDL)—a pathological molecule potentially involved in this relationship—could be affecting OA. The objectives of this study were to investigate the effect of hypercholesterolemia induced by high-fat diet (HFD) in cartilage from OA rabbits, and how oxLDL affect human chondrocyte inflammatory and catabolic responses.

**Design:** New Zealand rabbits were fed with HFD for 18 weeks. On week 6, OA was surgically induced. At the end of the study, cartilage damage and IL-1β, IL-6, MCP-1, MMP-13, and COX-2 expression in articular cartilage were evaluated. In addition, cultured human OA articular chondrocytes were treated with oxLDL at concentrations equivalent to those expected in synovial fluid from HFD rabbits, in the presence of IL-1β and TNFα. The effect of oxLDL on cell viability, nitric oxide production and catabolic and pro-inflammatory gene expression was evaluated.

**Results:** HFD intake did not modify cartilage structure or pro-inflammatory and catabolic gene expression and protein presence, both in healthy and OA animals. OxLDL did not affect human chondrocyte viability, ADAMTS5 and liver X receptor (LXR) α gene expression, but decreased the induction of IL-1β, IL-6, MCP-1, MMP-13, iNOS, and COX-2 gene expression and MMP-13 and COX-2 protein presence, evoked by cytokines.

**Conclusions:** Our data suggest that cholesterol intake *per se* may not be deleterious for articular cartilage. Instead, cholesterol *de novo* synthesis and altered cholesterol metabolism could be involved in the associations observed in human disease.

## Introduction

Osteoarthritis (OA) is one of the main causes of pain and disability, with high impact on life quality but also on national economies (1). It is a multi-factorial disease, and several risk factors like trauma, aging, gender, obesity and genetic predisposition have been associated with the development and progression of OA. In fact, new studies deepening in OA pathophysiology have revealed that this disease could be divided in different phenotypes (2). Among them, metabolic OA is of high importance. This phenotype arises from the wide association of OA with metabolic syndrome, with higher incidence of this condition in OA patients than in the population without the disease, and a more severe progression of OA in patients with metabolic syndrome (3).

Metabolic syndrome is defined by the presence of insulin resistance, obesity, hypertension and dyslipidaemia. Several studies have analyzed the role of each component in the development of OA, concluding that hypercholesterolemia and OA are significantly associated (4, 5). In addition, dysregulated lipid metabolism and cholesterol accumulation have been found in chondrocytes during OA (6, 7), and knockout mice with altered HDL metabolism have higher predisposition to the development of OA (8).

Despite the evidence showing a relationship between hypercholesterolemia and OA, epidemiological studies have found contradictory results about the use of statins to control circulating cholesterol levels and OA development. While a reduced progression of knee OA or lower progression of generalized OA in statin users have been reported (9, 10), no improvement of knee pain, joint function, or structural progression has been observed (11, 12). Therefore, there is insufficient data to prove an effect of circulating cholesterol levels on the onset and progression of OA.

Experimental models of OA allow to analyse the specific effect of dietary cholesterol in the development of the disease, disregarding other factors that are linked to hypercholesterolemia in humans. However, just a few studies have used this approach and obtained conflicting results (13–16). Since rabbits have been claimed as the best animal model for lipid-related research (17, 18), we employed rabbits with normal cholesterol metabolism in our study, in order to analyse the effect of high fat diet (HFD) on OA progression. Furthermore, we carried out an experimental model of high lipid intake that is not associated to an increase in weight gain, that allows to adequately address the contribution of added mechanical load and hyperlipidaemia in cartilage alterations (19–21).

Another suggested hypothesis is that oxidized low density lipoproteins (oxLDL) could promote inflammation during OA development, being the linking molecule between atherosclerosis and OA and explaining the association between this clinical complication of metabolic syndrome and OA (4). Different studies suggest that oxLDL could be deleterious for chondrocytes, inducing decreased cell viability and increased senescence (22–24), as well as hypertrophic-like changes (25). However, these studies did not asses these changes driven by oxLDL on an already inflamed environment such as that found in OA joints. Additionally, other studies suggest an anti-inflammatory and protective role of oxLDL in macrophages activated with LPS (26), as well as a proliferation-triggering effect of human quiescent fibroblasts (27).

Therefore, we aimed to investigate the effect of hypercholesterolemia induced by HFD in cartilage damage associated to OA in rabbits, and how oxLDL affect human chondrocyte inflammatory and catabolic responses.

## Materials and Methods

### Experimental Model in Rabbits

Four months old male New Zealand rabbits (*n* = 36) weighing 3–3.5 kg (Granja San Bernardo, Navarra, Spain) were used in accordance to local and European regulations on care and use of research animals, after approval of the protocol by the Institutional Ethics Committee and following ARRIVE guidelines.

The environmental and temperature conditions were kept constant throughout the study, with 12-h light and 12-h darkness cycles. Rabbits were kept in individual transparent 50 × 40 × 40 cm cages that allow social interactions. After 2 weeks of acclimatization with *ad-libitum* access to water and standard commercial chow, rabbits were randomly separated into four groups (20).

Sixteen randomly selected rabbits were fed HFD containing 0.5% cholesterol and 4% peanut oil (Sniff Spezialdiäten GmbH, Soest, Germany), and 20 rabbits received regular chow, both administered *ad-libitum*. Six weeks later, hypercholesterolemia was established and bilateral OA knee was induced in 10 randomly selected regular chow fed (OA group) and 10 randomly selected HFD animals (OA-HFD group) by anterior cruciate ligament transection (ACLT) and partial medial meniscectomy following an established protocol (20, 28). The surgery was performed on overnight fasted animals, in the morning, under general anesthesia by intramuscular injection of a combination of 2 mg/kg xylazine (Rompun, Bayer) and ketamine hydrochloride (Ketolar, Pfizer) in a 3:1 ratio. Procedures were carried out under aseptic conditions in an operating room. Antibiotic prophylaxis was performed with intramuscular cefonicid injections (100 mg/kg) (Smith K. Beecham) before surgery and the following 3 days. Six HFD animals (HFD group) and 10 regular chow animals (control group) did not undergo experimental intervention. Two OA-HFD animals died due to surgery complications. Blinding was performed by using a non-consecutive numerical code for animals in each group.

Our group has previously verified in several studies that the inflammation generated by the ACLT surgery does not affect the outcome of the joint injury (28). Therefore, we decided not to include a simulated surgery (Sham) group in this study to reduce the total number of animals used.

Body-weight was monitored at baseline and every other week throughout the study. Twelve weeks after OA induction, blood samples were collected from the marginal ear vein after overnight fasting and rabbits were then euthanized by intra-cardiac injection of Tiobarbital (B. Braun, Barcelona, Spain) 1 g/20 ml, following general anesthesia as previously described (28). Articular cavity was reached and left and right tibia cartilage samples were collected independently and immediately frozen to be used for molecular biology studies. Both femurs from each rabbit were also dissected and immersed in 4% paraformaldehyde for histological analysis.

As previously reported, feeding with this HFD did not induced weight gain, glucose metabolism alteration, or systolic blood pressure elevation (19, 20). However, these animals showed a significant increase both in circulating total cholesterol (control: 32 ± 10; HFD: 1,876 ± 287^*^; OA: 28 ± 4; OA-HFD: 2,050 ± 196^*^ mg/dl; ^*^p < 0.05 vs. control) and triglyceride concentration (control: 48 ± 7; HFD: 253 ± 97^*^; OA: 67 ± 12; OA-HFD: 290 ± 81^*^ mg/dl; ^*^p < 0.05 vs. control) (20).

### Histopathological Study

**Figure 1:**
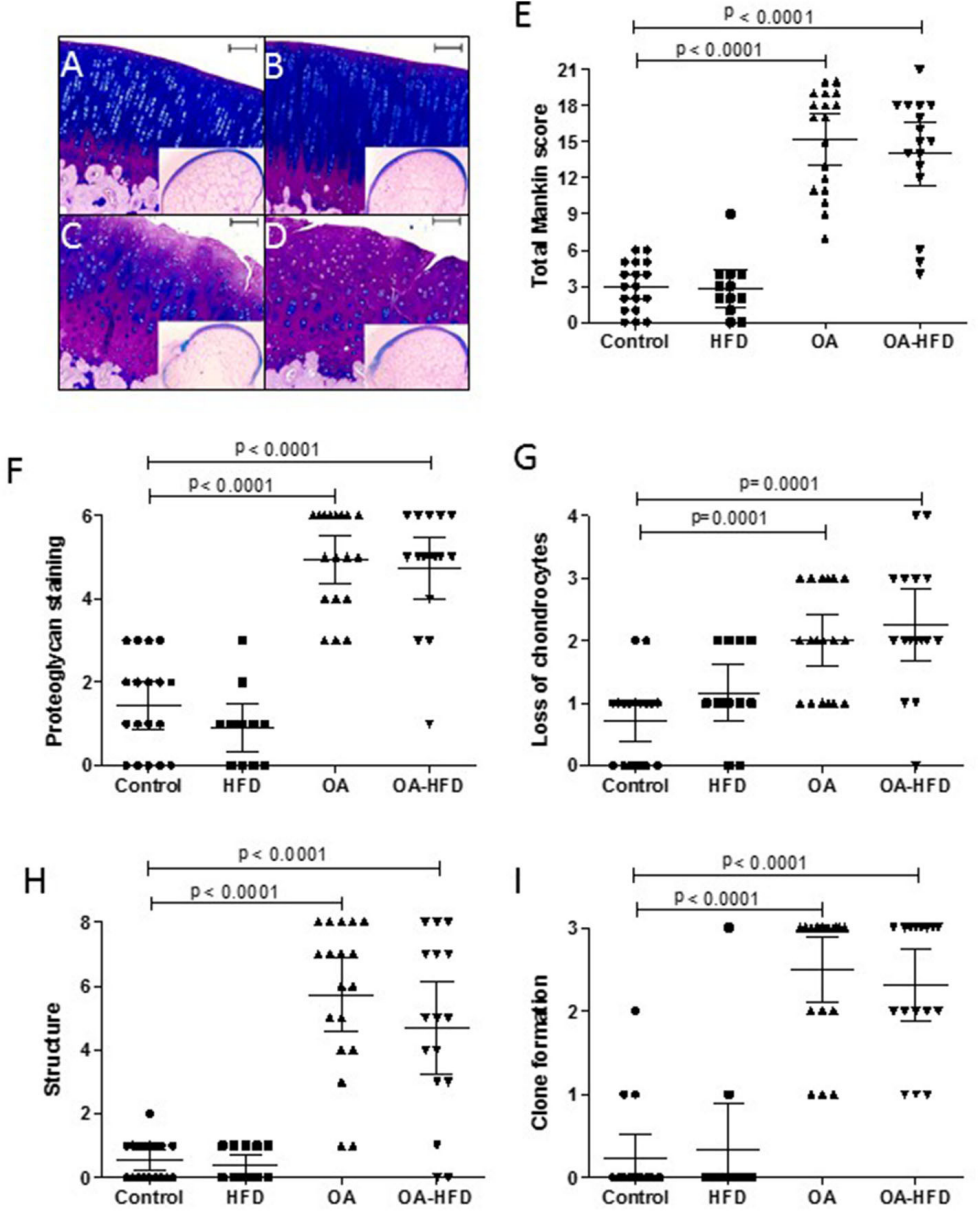
Histological characterization of cartilage damage. Representative sections of femur cartilage stained with Alcian-Blue PAS from control **(A)**, high fat diet (HFD) **(B)**, osteoarthritic (OA) **(C)**, and OA fed HFD **(D)** rabbits. An image of the whole femur section is presented in miniature. Scale bar = 100 μm. Histopathological changes in cartilage were measured using a modified Mankin score as described in Methods **(E)**, evaluating proteoglycan staining **(F)**, chondrocyte loss **(G)**, alteration of cartilage structure **(H)**, and clone formation **(I)**. Data are expressed as mean ± confidence interval; *n* = 12–18 samples per group.

After fixation in 4% paraformaldehyde, femurs were decalcified for 4 weeks in a solution containing 10% formic acid and 5% paraformaldehyde. The decalcified femoral condyles were cut in a sagittal plane along the central portion of the articular surface of each medial femoral condyle corresponding to the weight-bearing area, and then embedded in a paraffin block. Sections of 4 μm were stained with Alcian-Blue PAS to assess pathological changes in cartilage. These samples were evaluated using a modified version of Mankin's grading score system, which analyses four different parameters with a total score up to 21: cartilage structure (0–8), proteoglycan staining (0–6), loss of chondrocytes (0–4), and clone formation (0–3) (29). Two blinded observers evaluated the samples (AL-V and RL), and each data presented in Figure 1 was the mean for each sample between these observers.

### Immunohistochemical Studies

Immunohistochemical analyses were done in 4 μm femur cartilage samples. Antigen retrieval was performed by incubation with 0.1% trypsin from bovine pancreas (Sigma-Aldrich, USA) in 0.1% CaCl_2_. Inflammatory markers were visualized using anti-metalloproteinase (MMP)-13 (R&D systems, USA, MAB511, 30 μg/ml) and anti-ciclooxigenase (COX)-2 (sc-1745; Santa Cruz Biotechnology, USA, 1/100 dilution) antibodies as previously described (30). A secondary biotinylated anti-mouse and anti-goat IgG was used respectively for detection of positive signal through a horseradish peroxidase linked to an avidin/biotin complex (ABC) (Vector Laboratories, USA) using 3,3 diaminobenzidine tetra-hydrochloride as chromogen (Dako, Denmark). Sections were counterstained with Haematoxylin, dehydrated and mounted in DPX (Merck Millipore, USA). Positive immunoreactivity was evaluated in x20 magnification photographs obtained using a Leica DM3000 LED digital micro-imaging instrument (Leica Microsystems, USA). Each image was analyzed using the Image J software (National Institutes of Health, USA) and the percentage of positive area was calculated as previously described (30, 31).

### Human Chondrocyte Culture

Human chondrocytes were obtained from the FJD-Biobank (IIS-Fundación Jiménez Díaz, Madrid, Spain) and came from OA patients who underwent total knee replacement surgery. Cells were grown in Dulbecco's Modified Eagle Medium with 10% fetal bovine serum (FBS), 2 mM glutamine, and 100 U/ml of penicillin and streptomycin (Lonza, Belgium). At least four independent experiments were performed for each determination. Each replicate corresponds to an independent experiment carried out with chondrocytes extracted from a different donor. Cells were used within the first or second passage (32, 33).

### LDL Isolation and Cell Treatment

Human LDL were isolated from fresh plasma by sequential ultracentrifugation (34). After removing VLDL and IDL at 1.019 g/ml, LDL fraction between 1.019 and 1.063 g/ml was collected. LDL were then oxidized using 5 μM CuSO_4_ at 37°C for 2 h and the reaction was stopped by adding 0.3 mM EDTA. OxLDL were dialysed, sterile filtered and quantified using Bio-Rad protein assay (Bio-Rad Laboratories, USA). Then, they were diluted to 1 mg/ml with sterile PBS and stored at 4°C for no longer than 90 days. LDL from three different healthy donors were pooled after isolation, and then oxidized and quantified. Three different oxLDL pools were employed for all the experiments.

After 20 h in serum-free conditions, chondrocytes were treated with 10 or 40 μg/ml of oxLDL, in the presence or absence of 1 ng/ml of IL-1β and 10 ng/ml of TNFα (Peprotech, NJ, USA). Control cells were treated with LDL vehicle (PBS).

### Cell Viability Assay

Chondrocyte viability was tested using the methyl-thiazolyl-tetrazolium assay (MTT reagent, Sigma-Aldrich, MO, USA). Briefly, 7 × 10^3^ cells/well were plated in 96-well plates and treated as described above for 20 h. Then, the cells were incubated for 4 h with 10 μl/well of MTT (5 mg/ml). After formazan salt was dissolved with 10 mM HCl in 10% SDS, absorbance was measured at 570 nm.

### Nitrite Production

Nitrite accumulation was determined in the culture medium using the Griess reaction as previously described (33). Therefore, 4 × 10^4^ cells/well were seeded in 24-well plates and grown until confluence. Then, cells were treated as described above for 72 h. Nitrite content was evaluated in 50 μl of culture supernatant by incubating for 5 min with Griess reagent (equal volumes of 1% sulphanilamide in 5% phosphoric acid and 0.1% Naphtylethylenediamine–HCl) and absorbance was measured at 540 nm. All measurements were performed in duplicate.

### Gene Expression Analysis

Human primary chondrocytes were seeded in 6-well plates at 2 × 10^5^ cells/well and cultured until confluence. Then, cells were treated as described above for 24 h and lysed with Tripure Reagent (Roche, IN, USA). Rabbit cartilage samples were ground into a powder and mixed with Tripure Reagent (32).

RNA was isolated according to manufacturer's protocol and cDNA was synthesized using the High-Capacity cDNA Reverse Transcription Kit (Applied Biosystems, USA). TaqMan Gene expression assays were used to quantify mRNA expression in StepOnePlus™ detection system using StepOne™ software v2.2 (Applied Biosystems) (19, 34). HPRT was used as endogenous control and data was presented as relative expression to IL-1β and TNFα-stimulated chondrocytes or to healthy rabbits.

### Western Blot Studies

Briefly, 20 μg of total cell lysates were separated by 12% SDS–PAGE and transferred to a nitrocellulose membrane as described elsewhere (35). The following primary antibodies were applied: anti-COX-2 antibody (Santa Cruz Biotechnology; sc-1745; 1/100 dilution) and anti-MMP-13 antibody (Abcam; ab39012; 1/6000 dilution) in 3% BSA, overnight at 4°C. Antibody binding signal was detected by chemoluminescence through horseradish peroxidase-linked secondary antibodies. α-Tubulin (Sigma-Aldrich; T5168; 1/5000 dilution) was used for protein loading control. Densitometric measurements were normalized relative to protein presence in IL-1β and TNFα-stimulated chondrocytes using Quantity One software (Bio-Rad) (20).

### Statistical Analysis

Data obtained from each animal, articular tissue or *in vitro* experiments were analyzed using Kruskal-Wallis multiple comparison test followed by *post-hoc* analysis or Mann-Whitney tests, where appropriate, with Prism software (v5.01, GraphPad software, Inc). Results were expressed as mean ± confidence interval. *P* < 0.05 was considered statistically significant.

## Results

### Cartilage Damage in OA Rabbits Fed HFD

Histological parameters were assessed as previously described (29). At the end of the study, 18 Control, 12 HFD, 18 OA and 16 OA-HFD femurs were evaluated. Two control and two OA femurs could not be evaluated due to technical difficulties during sample processing.

The surgical induction of OA was successful in all knees of OA animals, with all the studied histological parameters significantly increased compared to non-OA animals (Figure 1). The administration of HFD did not modify the cartilage histology, with the HFD animals showing a score similar to control animals. In addition, OA-HFD animals showed the same damage than the standard chow fed OA rabbits (Figure 1).

### Molecular Changes in Cartilage From OA Rabbits Fed HFD

Considering the absence of worsening in cartilage histopathology in the presence of HFD, we decided to analyse molecular mediators of the OA process in cartilage from one tibia of each animal from the different study groups, to find out if hypercholesterolemia induced by HFD could be modifying molecular mediator expression in this tissue at a level not appreciable by histology.

**Figure 2:**
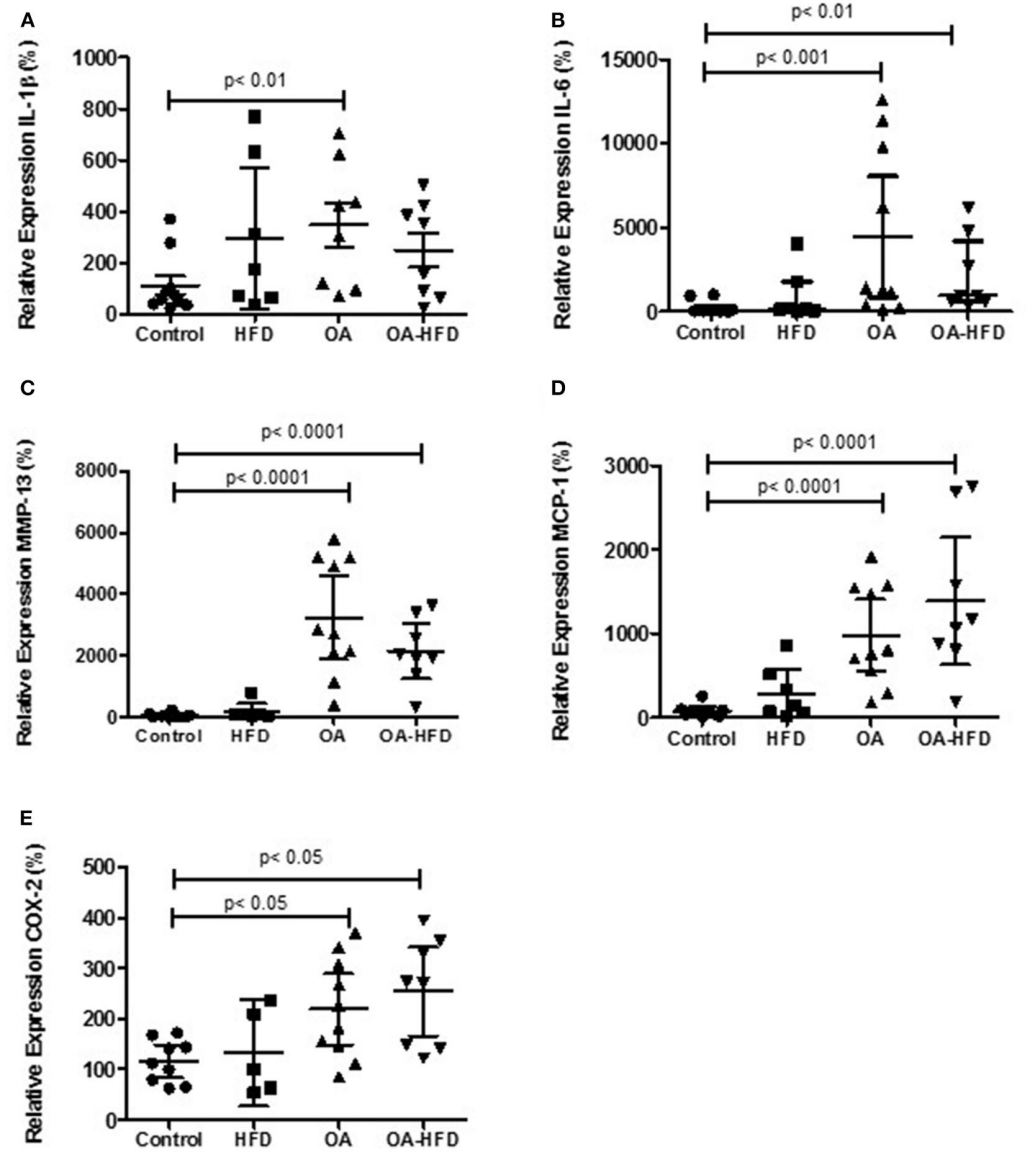
Differential gene expression examined by real time PCR in cartilage. IL-1β **(A)**, IL-6 **(B)**, MMP-13 **(C)**, MCP-1 **(D)**, and COX-2 **(E)** were measure in control, high fat diet (HFD), osteoarthritic (OA), and OA fed HFD (OA-HFD) rabbits. HPRT was used as endogenous control and results were expressed as fold change comparing to healthy rabbit. Data are expressed as mean ± confidence interval; *n* = 6–10 animals per group.

HFD did not modify IL-1β, IL-6, MMP-13, MCP1 nor COX-2 gene expression in comparison to Controls. OA animals showed a significant increased expression of these mediators (Figure 2). In OA-HFD animals, the expression of these pro-inflammatory and catabolic mediators was not significantly modified (Figure 2) in comparison to OA rabbits.

**Figure 3:**
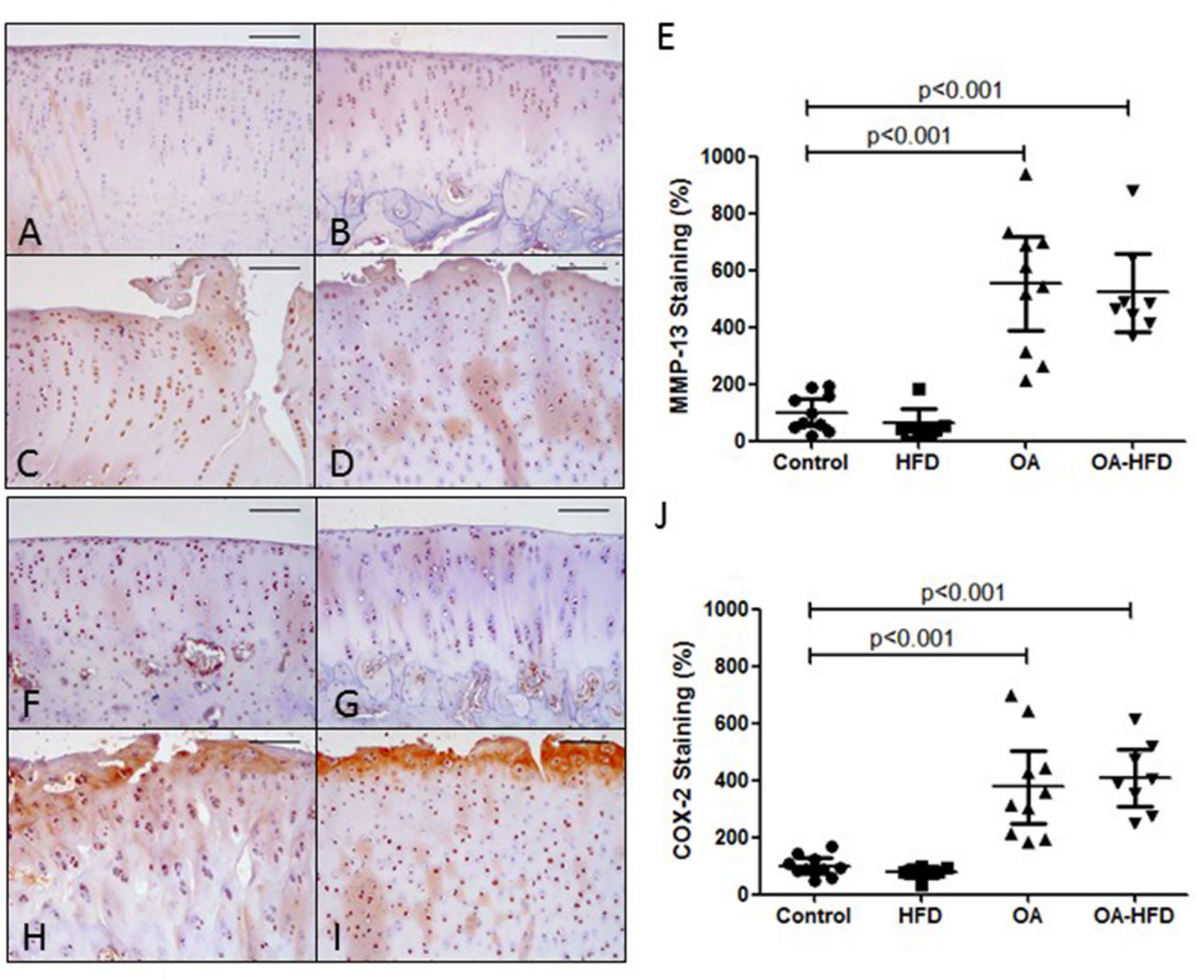
Inflammatory markers in rabbit cartilage. Representative sections of MMP-13 immunohistochemical staining in control **(A)**, high fat diet (HFD) **(B)**, osteoarthritic (OA) **(C)**, and OA fed HFD (OA-HFD) **(D)** femur cartilage, and MMP-13 staining cuantification **(E)**. Representative sections of COX-2 immunohistochemcal staining in control **(F)**, HFD **(G)**, OA **(H)**, and OA-HFD **(I)** femur cartilage, and COX-2 staining cuantification **(J)**. Scale bar = 100 μm. Results were expressed as fold change comparing to healthy rabbit. Data are expressed as mean ± confidence interval; *n* = 7–10 animals per group.

We also tested the presence of MMP-13 and COX-2 proteins in the articular cartilage of the rabbits. As can be observed in Figure 3, the tissue staining of both proteins was significantly enhanced in the cartilage of OA and OA-HFD when compared to controls (Figures 3E,J), although the tissue localization for these proteins was similar between these two groups. Furthermore, OA-HFD rabbits showed no differences in the intensity of the staining for MMP-13 and COX-2 when compared to OA animals (Figures 3C–E,H–J).

### Effect of oxLDL on Human Chondrocyte Viability

Given that oxLDL have been suggested as a potential link between metabolic syndrome complications and OA (4), we decided to study the effect of oxLDL on chondrocytes during an OA-like inflammatory environment. The selected doses were in the range of oxLDL levels expected in the synovial fluid of our hypercholesterolemic rabbits in accordance with published data (36–38).

**Figure 4:**
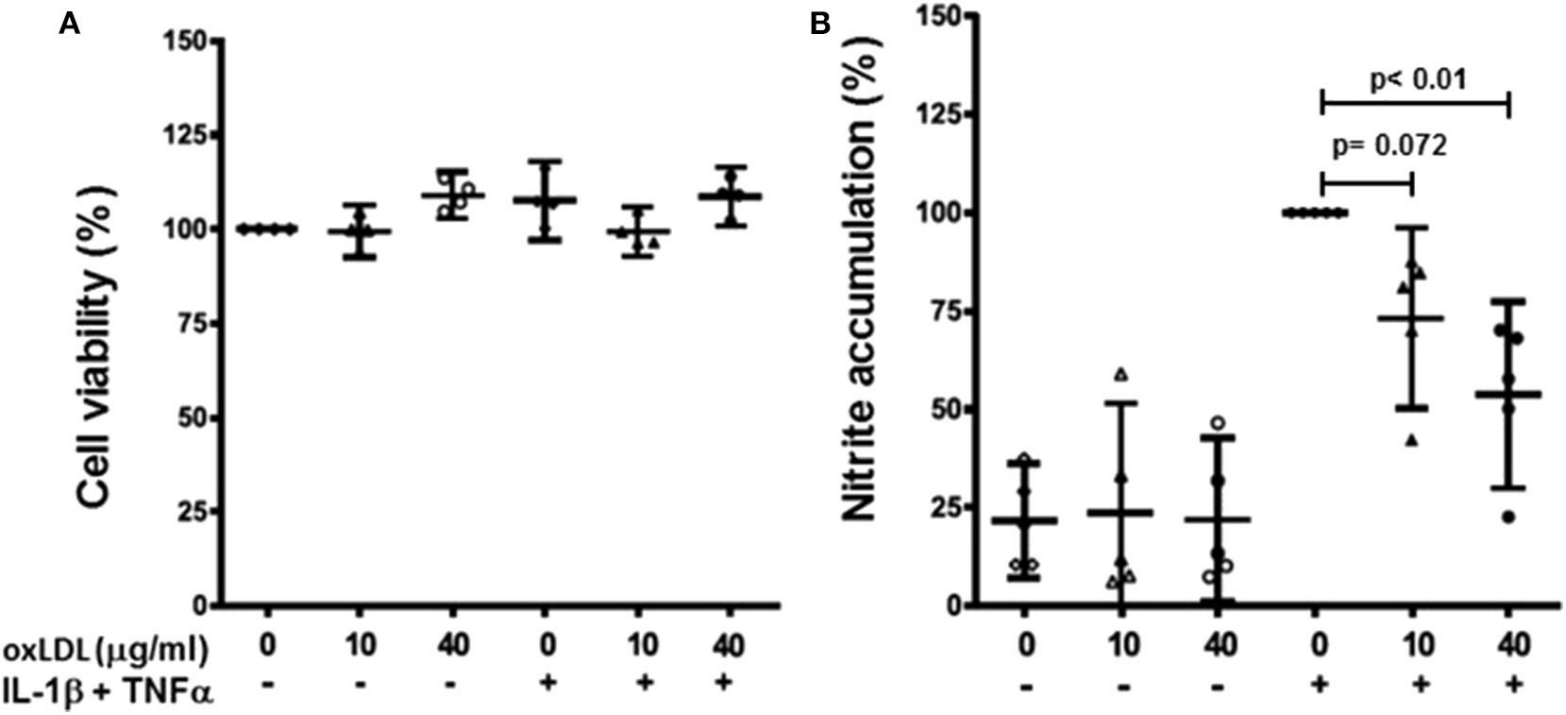
Effect of oxLDL on human chondrocyte viability and nitric oxide production. Human chondrocytes were cultured in the presence or absence of IL-1β and TNFα with 10 and 40 μg/ml of oxLDL. Cell viability was measured using MTT assay **(A)** and nitric oxide production was evaluated as nitrite accumulation in culture medium after 72 h using Griess reaction **(B)**. Data are expressed as mean ± confidence interval. *n* = 4 experiments for cell viability, and *n* = 5 for nitrite accumulation (replicates employing different tissue donors).

Human primary chondrocytes were stimulated with the selected concentrations of oxLDL in the presence or absence of IL-1β and TNFα pro-inflammatory stimuli. The concentrations used in this study did not affect chondrocyte viability as measured by MTT assay (Figure 4A).

### Effect of oxLDL on Pro-inflammatory and Catabolic Mediator Expression in Chondrocytes

As they did not affect chondrocyte viability, we studied the effect of the oxLDL on different pro-inflammatory and catabolic mediators produced by human OA primary chondrocytes.

Firstly, we measured nitric oxide (NO) production as nitrite accumulation in culture media. Both 10 and 40 μg/ml of oxLDL reduced the production of NO induced by pro-inflammatory stimuli in human primary chondrocytes, and they did not affect NO production in non-stimulated cells (Figure 4B).

**Figure 5:**
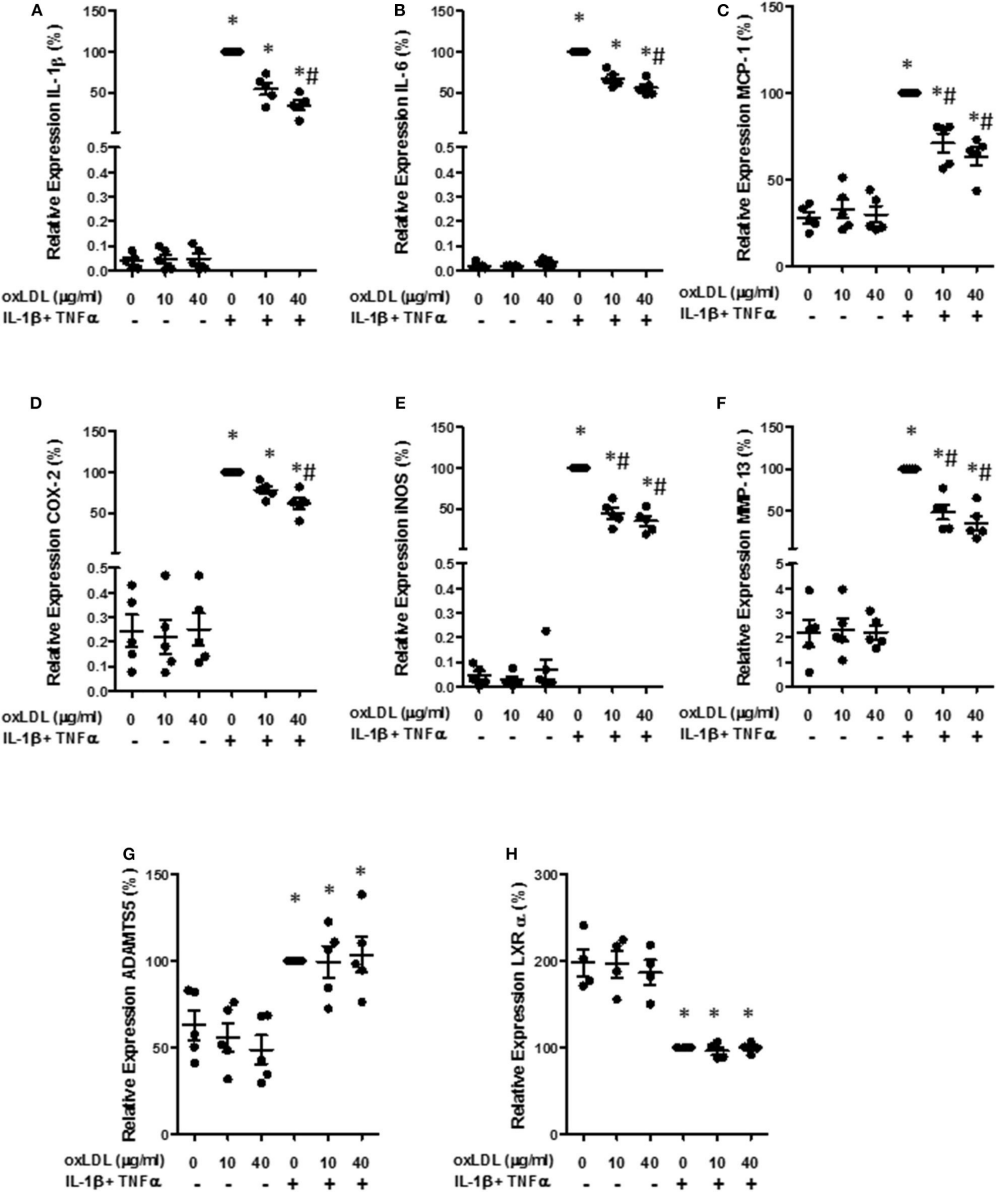
Effect of oxLDL on inflammatory and catabolic mediator expression in human chondrocytes. Human chondrocytes were cultured in the presence of IL-1β and TNFα and 10 or 40 μg/ml of oxLDL, and IL-1β **(A)**, IL-6 **(B)**, MCP-1 **(C)**, COX-2 **(D)**, iNOS **(E)**, MMP-13 **(F)**, ADAMTS5 **(G)**, and LXRα **(H)** gene expression was analyzed using real time PCR. HPRT was used as endogenous control and results were calculated as a percentage over IL-1β and TNFα-stimulated control. Data are expressed as mean ± confidence interval. *p < 0.05 vs. basal medium; ^#^*p* < 0.05 vs. IL-1β+TNFα. *n* = 4–5 replicates, employing different donors.

Then, we quantified gene expression of different mediators in human chondrocytes. The presence of oxLDL did not significantly modify the gene expression of the different mediators studied. However, the induction of IL-1β, IL-6, and MCP-1 gene expression by a pro-inflammatory cocktail of IL-1β and TNFα was significantly reduced in the presence of both 10 and 40 μg/ml of oxLDL (Figures 5A–C). We also analyzed the expression of inducible NO synthase (iNOS) and COX-2, as they are important enzymes for the production of pro-inflammatory mediators, and oxLDL also decreased the expression of these enzymes (Figures 5D,E). In addition, we analyzed the expression of catabolic mediators. MMP-13 gene expression was significantly decreased by oxLDL, while ADAMTS5 was not modified by the presence of the lipid (Figures 5F,G).

**Figure 6:**
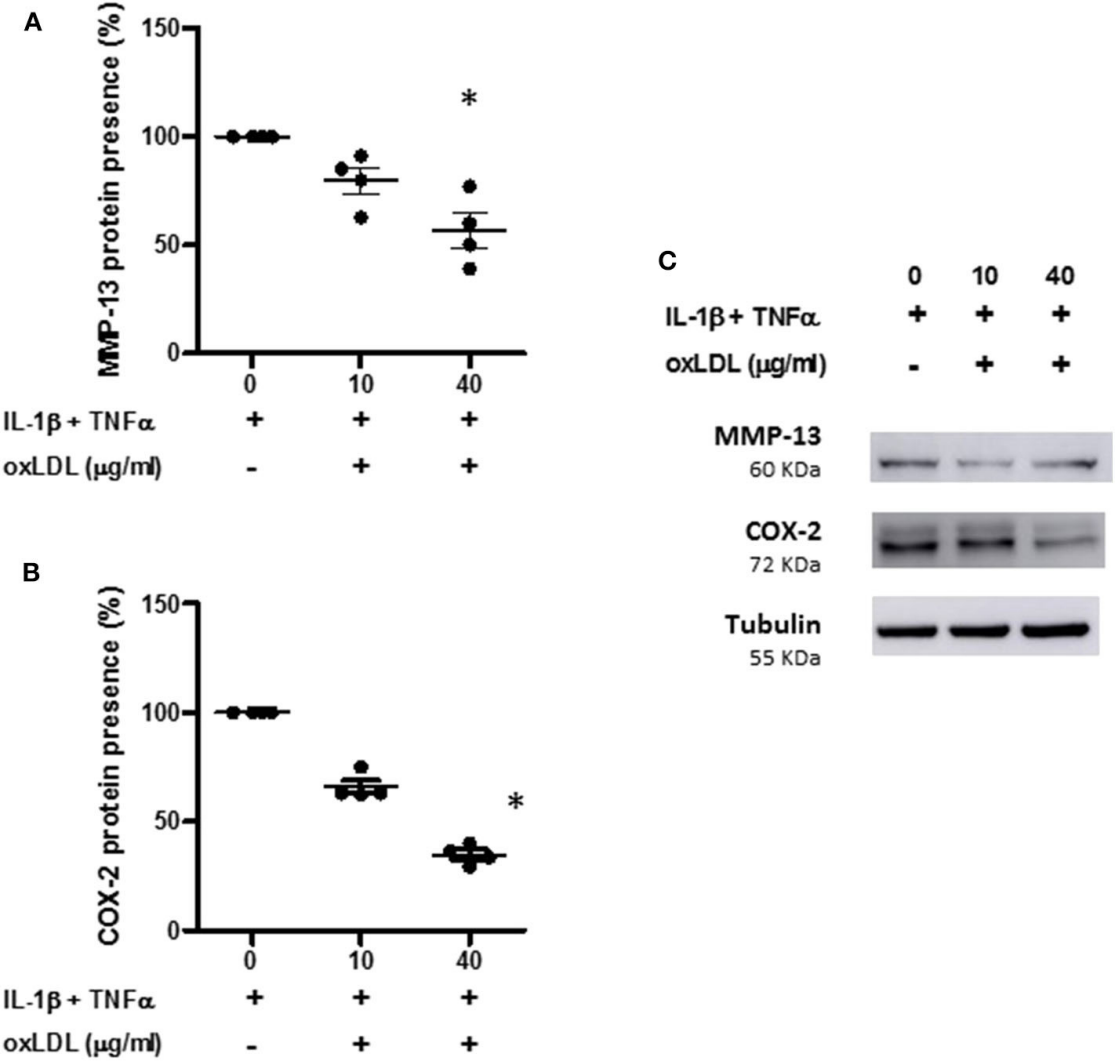
Effect of oxLDL on MMP-13 and COX-2 protein presence in human chondrocytes. Human chondrocytes were cultured in the presence of IL-1β and TNFα and 10 or 40 μg/ml of oxLDL, and MMP-13 **(A)** and COX-2 **(B)** protein expression was analyzed by Western-blot experiments in total cell proteins. Tubulin was used as protein loading control and results were calculated as a percentage over IL-1β and TNFα-stimulated control. Data are expressed as mean ± confidence interval. *p < 0.05 vs. IL-1β+TNFα. *n* = 4 replicates, employing different donors. A representative Western blot is shown in **(C)**.

We also measure the protein presence of MMP-13 and COX-2 in the cell extracts from human OA chondrocytes incubated with the pro-inflammatory cocktail in the presence or absence of oxLDL. As can be observed in Figure 6, 40 μg/ml of oxLDL was able to significantly decrease the synthesis of these proteins induced by cytokines (Figures 6A–C).

### Effect of oxLDL on Liver X Receptor (LXR) α in Chondrocytes

Different anti-inflammatory effects of oxLDL have been attributed to LXRα activation. Therefore, we measured the gene expression of this nuclear receptor, that is able to auto-regulate its own expression (39). As can be observed in Figure 5H, the incubation with the cocktail of cytokines significantly diminished the gene expression of LXRα, although no significant effect was observed with the addition of oxLDL.

## Discussion

The present study shows that hypercholesterolemia induced by HFD does not alter knee cartilage structure in rabbits. In fact, HFD has no effect on cartilage histopathological score and OA-associated gene expression and protein presence after surgically-induced OA. Accordingly, oxLDL does not modify human articular chondrocyte viability, and decreases the expression of pro-inflammatory and catabolic factors under inflammatory conditions *in vitro*.

Experimental models of OA allow to analyse the specific effect of dietary cholesterol in the development of the disease, disregarding other factors that are linked to hypercholesterolemia in humans. However, just a few studies have used this approach. Two studies analyzed the effect of HFD in mice with altered cholesterol metabolism (15, 40), and found that exposure to high cholesterol levels increased spontaneous cartilage damage in healthy animals. A recent work showed that even wild-type Wistar rats presented OA-like changes when fed HFD (16). However, de Munter et al. observed that HFD did not worsen cartilage structure in wild-type mice and in transgenic animals with altered cholesterol metabolism after OA induction (13, 14). In our study, rabbits with normal cholesterol metabolism were used to analyse the effect of HFD on OA progression.

As mentioned before, rabbits have been claimed as the best animal model for lipid-related research (18). In contrast to mice and rats, rabbits have a lipoprotein metabolism similar to humans, where the main cholesterol pool is from a hepatic origin and LDL is the predominant plasma lipoprotein (18). The apoenzyme (Apo) profile and the hepatic LDL receptors are also similar in rabbits and humans (17, 41). In contrast to rodents, rabbits present cholesteryl ester transfer protein (CETP) activity, which is also active in humans and essential for HDL metabolism (18, 42). Higher activity of this enzyme has been associated with anti-inflammatory properties, reduced adipogenesis and lower body mass index, and may also be involved in reducing circulating oxLDL levels. In fact, pharmacological inhibition of CETP has been linked to increased sepsis and mortality (43). These similarities between rabbits and humans suggest that rabbits are a better model to analyse cholesterol effects on OA than mice and rats used in previous studies.

Results found in the present study using a rabbit model for diet-induced hypercholesterolemia suggest that under normal cholesterol metabolism, external cholesterol intake does not affect cartilage structure. These data may reflect human hypercholesterolemia associated with bad eating habits in the absence of altered cholesterol metabolism more strictly than rodent models. Inconsistent results from epidemiological studies trying to link statin use and cholesterol levels with OA might be associated with the results showed in this study (9–12). In fact, several articles have described an anti-inflammatory and anti-catabolic effect of statins in cartilage *in vivo* and *in vitro*, which is independent of their cholesterol lowering properties (44–46). This protective effect has been associated with inhibition by statins of protein prenylation during cholesterol biosynthesis, which is able to regulate collagenase expression and cartilage breakdown in chondrocytes and cartilage explants (47, 48). This way, statins have been shown to reduce spontaneous cartilage damage in aging mice (49) and OA development in several animal models of the disease (44, 50–52). These data suggest that cholesterol biosynthesis, instead of cholesterol intake, may be relevant to cartilage metabolism, supporting the absence of a direct effect of HFD on cartilage damage progression in our study.

OxLDL may be produced in an inflammatory environment such as the arthritic joint (53), and they have been suggested to be a key player in the relationship between atherosclerosis, a clinical complication of metabolic syndrome, and OA (4). It has been previously demonstrated that human OA cartilage express LOX-1, indicating that this tissue is able to respond to oxLDL (22, 54). It has been reported that chondrocytes treated with oxLDL showed decreased cell viability and increased senescence (22–24, 54), as well as hypertrophic-like changes (25). However, we observed that oxLDL stimulation did not affect human primary chondrocyte viability, and even decreased pro-inflammatory and catabolic mediator expression induced by IL-1β and TNFα. LOX-1 expression in chondrocytes has been shown to be disease-specific (22, 54) and LOX-1 levels increase in human OA cartilage (55). The expression of this receptor and, therefore, the ability of chondrocytes to respond to oxLDL could also be dependent on the inflammatory state. In the same sense, other studies have demonstrated similar protective roles of oxLDL by decreasing the pro-inflammatory state, in a model of macrophages activated with LPS (26). This fact could determine how chondrocytes behave after oxLDL treatment in the different studies where it has been assessed. In addition, it has been shown that the degree of oxidation may have different effects on cells. In osteoblasts, mild oxLDL did not decrease cell viability and even increased proliferation, while standard oxLDL at high concentrations decreased cell viability and proliferation (56). Regarding the degree of oxidation, in an independent study Zettler and collaborators showed that a stimulation of oxLDL increased cell proliferation in quiescent human and rabbit fibroblasts, through the up-regulation of regulatory proteins of the cell cycle (27). In contrast to other published works, our study uses LDL that have been mildly oxidized by reducing the time of Cu^2+^ treatment. This means that these oxLDL better reflect the joint environment, as it has been reported that, even in a high inflammatory environment like that observed in rheumatoid arthritis joints, LDL are mildly oxidized in synovial fluid (53). Therefore, different species and degree of LDL oxidation could account for the differences observed between experiments.

To our knowledge, this is the first study using mild oxLDL to treat human primary chondrocytes that are stimulated by pro-inflammatory cytokines associated to the OA environment. Our results suggest that the oxLDL that could be present in OA synovial fluid may not be detrimental for cartilage, and even have anti-inflammatory properties, which might be a compensatory response to counteract the inflammatory environment. In fact, activation of LXR in human OA chondrocytes has been reported to have anti-catabolic effects under inflammatory stimulation (57). In murine chondrocytes, LXR induction with agonists decreases ADAMTS4, MMP-2, and MMP-13 gene expression (58). OxLDL have been shown to have a biphasic effect on NF-κB activation, depending on cell type and incubation time. Oxysterols, which are thought to be at least partially responsible for the biological effect of oxLDL, are able to inhibit NF-κB activation by pro-inflammatory agents, probably through LXRα (59). Oxysterols trigger both LXRα and LXRβ activation, inducing their target genes, which control lipid synthesis and metabolism, such as ApoE, CETP, ABCA1 (ATP Binding Cassette A1) transporter, ABCG1 transporter, and LXRα (39). We tested whether mildly oxLDL could be triggering an anti-inflammatory response through LXRα, as the activation of this nuclear receptor is able to induce its own gene expression (39). According to our data, oxLDL presence was not able to modify the decrease in LXRα expression induced by cytokines. These data suggest that the anti-inflammatory effect of oxLDL could not be mediated by LXR activation. However, these effects, which have been attributed to the inhibition of NF-κB activation, could be exerted by other bioactive oxidized lipids that are present in oxLDL, such as oxidized phospholipids, oxidized poly unsaturated fatty acids or different aldehydic products (59). Therefore, further studies are needed in order to identify the oxLDL component that could drive a protective effect on OA progression.

In contrast to the *in vitro* results, pro-inflammatory gene expression in cartilage from rabbits was not reduced by the increased cholesterol levels induced by HFD. Very recently, de Munter et al. observed that oxLDL uptake by synovial macrophages prevented other cell types from being activated by this lipid (60). These results suggest that, in our model, synovial macrophages could hoard the available oxLDL and impede their anti-inflammatory actions on cartilage.

In conclusion, the present study shows that, on an *in vivo* model of hypercholesterolemia in rabbits, cholesterol intake does not worsen cartilage degeneration and neither promotes structural and histopathological changes in rabbits with OA. These results are consistent with our *in vitro* model, where mildly-oxidized LDL do not show lipotoxic effects on human articular chondrocytes; conversely, they decrease the expression of pro-inflammatory and catabolic factors under inflammatory conditions. Therefore, these data suggest that cholesterol *per se* may not be deleterious for knee cartilage; instead, the effect of this molecule on OA progression might derive from secondary processes occurring during *de novo* biosynthesis, as suggested by previous studies using statins to inhibit this pathway.

## Data Availability Statement

The raw data supporting the conclusions of this article will be made available by the authors, without undue reservation.

## Ethics Statement

The animal study was reviewed and approved by Animal Welfare Committee IIS-Fundacion Jimenez Diaz.

## Author Contributions

AV: design of the study, acquisition, analysis, interpretation of data, and drafting the article. AL-V: acquisition, analysis, interpretation of data, and drafting the article. AL: acquisition, analysis of data, literature revision, and data discussion. SP-B: acquisition of data. AL-R: literature revision and data discussion. GH-B and RL: conception, design of the study, interpretation of data, and drafting the article. All authors critically revised the manuscript for important intellectual property and approved the final version to be submitted.

## Conflict of Interest

The authors declare that the research was conducted in the absence of any commercial or financial relationships that could be construed as a potential conflict of interest.
